# Body mass index and 12-year mortality among older Mexican Americans aged 75 years and older

**DOI:** 10.1186/s12877-022-02945-4

**Published:** 2022-03-21

**Authors:** Reshma Jadhav, Kyriakos S. Markides, Soham Al Snih

**Affiliations:** 1 School of Medicine, University of Texas Medical Branch, Galveston, TX USA; 2 Department of Preventive Medicine and Population Health, University of Texas Medical Branch, Galveston, TX USA; 3 Sealy Center on Aging, University of Texas Medical Branch, 301 University Blvd., Galveston, TX USA; 4 Department of Nutrition, Metabolism, and Rehabilitation Sciences/School of Health Professions, University of Texas Medical Branch, Galveston, TX USA; 5 Division of Geriatrics & Palliative Medicine /Department of Internal Medicine/School of Medicine, University of Texas Medical Branch, Galveston, TX USA

**Keywords:** Obesity, Mortality, Oldest old, Mexican American, Hispanic

## Abstract

**Background:**

The role of obesity in mortality in the very old and old-oldest Hispanic population has not been studied. The objective of this study was to examine the effect of body mass index (BMI) on 12-year mortality among older Mexican Americans aged 75 years and older.

**Methods:**

Twelve year prospective cohort study consisting of a population-based sample of 1415 non-institutionalized Mexican American men and women aged 75 and older from 5 southwestern states: Arizona, California, Colorado, New Mexico, and Texas. Data was from Wave 5 of the Hispanic Established Population for the Epidemiologic Study of the Elderly (2004/2005–2016). Socio-demographics, body mass index (BMI), self-reported medical conditions, disability, depressive symptoms, falls, Mini-Mental-State-Examination (MMSE), and Short Physical Performance Battery (SPPB) were assessed at baseline during 2004–2005. BMI (Kg/m^2^) was classified as underweight (< 18.5), normal weight (18.5 to < 25), overweight (25 to < 30), obesity category I (30 to < 35), and obesity category II/morbid obesity (≥ 35). For assessment of mortality, deaths were ascertained through the National Death Index and report from relatives. Cox proportional hazards regression analysis was performed to estimate the hazard ratio (HR) of 12-year mortality as a function of BMI categories at baseline.

**Results:**

The mean BMI was 27.5 ± 1.7 with participants classified as 1.8% underweight, 30.8% normal weight, 39.2% overweight, 20.7% obesity category I, and 7.6% obesity category II/morbid obesity. Mexican Americans aged ≥75 years with overweight or obesity category I had a reduced HR of death (0.82, 95% CI = 0.70–0.96 and 0.75, 95% CI = 0.62–0.91, respectively) over 12-years of follow-up. The HR of death for underweight and obesity category II/morbid obesity participants was 1.59 (95% CI = 1.03–2.45) and 1.12 (95% CI = 0.85–1.46), respectively. Female participants and those with high scores in the MMSE and SPPB had decreased risk of death.

**Conclusions:**

This study showed the protective effect of overweight and obesity on mortality in Mexican Americans above 75 years of age, which might have implications when treating older adults with overweight and obesity.

## Background

Obesity continues to rise in the United States. According to the Centers for Disease Control and Prevention (CDC), the prevalence of obesity in the U.S. has increased from 30.5 to 42.4% from 1999 to 2000 to 2017–2018 [1]. The Hispanic population is affected by the obesity epidemic, with 42.5% classified as obese compared with non-Hispanic white (34.5%), non-Hispanic black (48.1%), and non-Hispanic Asian adults (11.7%) [1]. Overall, the prevalence of obesity among women (38.3%) is higher than among men (34.3%) [1]. U.S. born Hispanics (47.1%) have a higher prevalence of obesity than foreign-born Hispanics (36.3%) [2].

A strong association has been found between obesity and mortality in several longitudinal studies in young and middle-aged adults [3]. However, an “obesity survival paradox” is seen in older persons above 65 years in which an increased risk in all-cause mortality from obesity is not seen, and the optimal body mass index (BMI) is increased to between 24 and 35 kg/m^2^, with most in the range of 27 to 30 kg/m^2^ [4]. Several studies have reported a decreased association of overweight (25 - < 30 kg/m^2^) [5–13] and obesity (> 30 kg/m^2^) [5–9, 11, 13, 14] with mortality compared to normal weight in older persons above 65 years and older.

The protective effect of obesity on mortality that has been reported in adults aged 65 years and older has also been reported in the very old and the oldest-old adults. For example, Kuznicka et al. using the PolSenior study found that a BMI of 25.0 to 34.9 kg/m^2^ was associated with the lowest mortality and underweight (BMI < 18.5 kg/m^2^) was associated with the highest mortality (*p* < 0.001) among those 65–79 years of age, while for those aged 80 year and older a BMI of 25.0–39.9 kgm^2^ was associated with lowest mortality while BMI ≤ 24.9 kg/m^2^ was associated with highest mortality (*p* < 0.001) over 3 years of follow-up [15]. In another study, Stenholm et al. found in a cohort of Finish older adults aged 50–91 years that those aged 70 years and older who were overweight and obese had 23 and 24%, respectively, decreased risk of mortality over 33 years of follow-up [16]. Dahl et al. found in a cohort of Swedish older adults aged 70 to 95 years that those in the overweight group had a 20% decreased risk for mortality compared to the normal group over 4 years of follow-up for those younger than 80 years and 2 years of follow-up for those over 80 years [17]. Wang et al. using the Chinese Longitudinal Healthy Longevity Survey (CLHLS) found that participants aged 80 years and older with overweight and obesity had a 11 and 9% decreased risk of mortality compared with the normal weight group over 3 years of follow-up [18].

Between now and 2050, those who are 65 years and older in the Hispanic population are expected to experience rapid aging [19]. By 2050, the percentage of Hispanics aged 65 and older is predicted to grow by 134% while the percentage of Non-Hispanic Whites is predicted to grow 58.4% [19, 20]. At birth in the United States, the life expectancy for Hispanics (78.83 years for males, 84.05 for females) is longer than for non-Hispanic whites (76.30 years for males, 81.06 for females) and non-Hispanic Blacks (71.41 for males, 77.62 for females) [21]. Male Mexican Americans live about 2.5 years and 7.4 years longer than their non-Hispanic white and non-Hispanic black counterparts, respectively [21]. Female Mexican Americans live about 3 years and 6.5 years longer than their non-Hispanic white and non-Hispanic black counterparts, respectively [21]. According to the National Vital Statistics report, the Hispanic population has a ‘survival advantage’ by age 65 compared to other populations in the U.S. and it increases with age [21]. Approximately, 87.7% of the Hispanic population survive to age 65 compared to 84.5% of the non-Hispanic white population and 76.5% of the non-Hispanic black population [21]. This report also found that 50.7% of the Hispanic population survive to age 65 compared with 41.5% in the Non-Hispanic White population and 32.1% in the non-Hispanic black population [21].

The role of obesity in mortality in the very old and old-oldest Hispanic population has not been studied. Therefore, we examined BMI as predictor of 12-year mortality among Mexican American older adults enrolled in the Hispanic Established Epidemiologic Study of the Elderly (HEPESE) aged 75 years and older. We hypothesize a protective effect of overweight and obesity on mortality in Mexican Americans above 75 years of age.

## Methods

### Sample

Data were from the HEPESE, a longitudinal cohort study of non-institutionalized Mexican Americans 65 years and older who reside in five southwestern states of the U.S. (Arizona, California, Colorado, New Mexico, and Texas). The original HEPESE cohort included 3050 Mexican Americans aged 65 years and older who were interviewed in-home face to face or via proxy in 1993/94. In 2004/05 a new cohort of 902 participants aged 75 years and older were added to the 1167 participants of the original cohort who were 75 years and older (*N* = 2069). Bilingual interviewers were trained to gather information on socio-demographic, health conditions, and psycho-social characteristics of respondents in their language of choice every 2 or 3 years. Nine waves of data have been collected. Information and data for the HEPESE are available at the National Archive of Computerized Data on Aging [22]. The present study used data collected from Wave 5 (hereafter referred as baseline) to Wave 9 (2004/05–2016), allowing for approximately 12 years of follow-up data. Out of 2069 eligible participants, we excluded 653 with incomplete information on body mass index and covariates. The final analytical sample included 1415 participants. Excluded participants were significantly more likely to be older, unmarried or US-born; to have a lower level of education, lower short physical performance (SPPB) and lower Mini-Mental State Examination (MMSE) scores; and to report more diabetes, high depressive symptoms, any or more falls, and limitations in activities of daily living (ADLs) than included participants. At the end of follow-up (2016), 376 participants were re-interviewed in person, 140 were lost to follow-up, and 899 were confirmed dead through the National Death Index and report from relatives.

### Measurements

#### Predictor variable

Baseline measured body mass index (BMI) was calculated as weight in kilograms divided by height in squared meters. BMI was grouped according to the National Institutes of Health obesity standards (< 18.5 Kg/m^2^, underweight; 18.5 to < 25 Kg/m^2^, normal weight; 25 to < 30 Kg/m^2^, overweight; 30 to < 35 Kg/m^2^, obesity category I; and ≥ 35 Kg/m^2^, category II/morbid obesity).

#### Outcome

All cause mortality over 12 years of follow-up. Deaths were ascertained through the National Death Index and report from relatives.

#### Covariates

Sociodemographic variables (age, sex, marital status, years of formal education, and marital status), current smoking, and self-reported medical conditions (hypertension, diabetes, stroke, cancer, heart attack, or hip fracture). Depressive symptoms were measured with the Center for Epidemiologic Studies Depression Scale. The scale consists of 20 items that ask how often specific symptoms were experienced in the past week and were scored on a 4-point scale (ranging from rarely/none of the time to most/all the time: 0, 1, 2, 3) and total scored range 0–60 [23]. A score of 16 or greater is used to determine a clinical range for those with depressive symptoms [24]. Cognitive function was assessed with the Mini Mental Status Examination (MMSE), with scores ranging from zero to 30. MMSE screens for difficulties in orientation, working memory, naming, language, image copying, and episodic memory [25]. Fall status was established by asking participants “During the last 12 months, how many times did you fall and land on the floor or ground?” and categorized as ≥1 falls in the last 12-months. Functional disability was assessed using seven items (walking across a small room, bathing, grooming, dressing, eating, transferring from a bed to a chair, and using the toilet) from a modified version of the Katz ADL scale [26]. Physical function was assessed using the SPPB, which includes three lower body extremity tests (walking speed, standing balance and repeated chair stands. Scores range from 0 to 12 with higher scores indicating higher physical functioning [27].

### Statistical analysis

ANOVA, Chi square, and Fisher exact tests were performed to describe the baseline sample characteristics by BMI category. Cox proportional hazards regression analysis was performed to estimate the hazard ratio of 12-year mortality as a function of baseline BMI category controlling for all covariates. Those subjects who died or were unable to be located were censored at the date of last follow-up (last interview date for the 12-year follow-up). Three models were performed. Model 1 included BMI categories with all covariates. Model 2 was not adjusted for comorbidities. In Model 3, participants who died the first 2 years of follow-up and those were current smokers were excluded. The Cox proportional hazard regression assumption was confirmed. Statistical analysis was performed using the SAS, version 9.4 (SAS Institute, Inc., Cary NC).

## Results

Table 1 presents the descriptive characteristics of the overall sample and by BMI categories. The overall mean age was 81.3 ± 4.5 years. The mean BMI was 27.5 ± 1.7 with participants classified as 1.8% underweight, 30.8% normal weight, 39.2% overweight, 20.7% obesity category I, and 7.6% category II/morbid obesity. Sixty percent of the participants were female, 45.3% were married, 58.0% were born in the United States, and the mean years of education was 5.5 ± 3.9. Mean MMSE and SPPB scores were 22.8 ± 5.4 and 6.4 ± 3.4, respectively. Sixty-two percent of participants reported hypertension, 8.0% heart attack, 6.9% cancer, 31.9% diabetes, 5.9% stroke, and 14.7% had high depressive symptoms at baseline. Thirty-two percent of participants had more than one fall in the past year and 24.6% had ADL disability at baseline. Sixty-four percent % of participants died over 12 years of follow-up. Female participants were significantly more likely to be in the obesity category I or obesity category II/morbid obesity. Overweight participants were significantly more likely to be married and to have higher level of education than those in other BMI categories. Underweight participants were significantly more likely to be current smokers and to have lower scores on the MMSE than those in the other BMI categories. Participants with category II/morbid obesity had the lowest mean years of education, scored lower in the SPPB, and were more likely to report hypertension, diabetes, and ADL disability compared to those in other BMI categories.

**Table 1:** Baseline descriptive characteristics of the sample by BMI categories (*N* = 1415)

BMI Categories							
Variables	Total N (%)	Underweight N (%)	Normal Weight N (%)	Overweight N (%)	Obesity category I N (%)	Obesity category II/Morbid Obesity N (%)	***P***-value
**Total**	1415 (100)	25 (1.8)	436 (30.8)	554 (39.2)	293 (20.7)	107 (7.6)	–
**Age, mean (SD)**	81.3 ± 4.5	83.8 ± 5.6	82.3 ± 5.1	80.9 ± 4.2	80.7 ± 4.4	79.6 ± 3.3	0.2769
**Gender**							
Male	561 (39.7)	10 (40.0)	176 (40.4)	248 (44.8)	103 (35.2)	24 (22.4)	0.0002
Female	854 (60.4)	15 (60.0)	260 (59.6)	306 (55.2)	190 (64.9)	83 (77.6)	
**Married**	641 (45.3)	11 (44.0)	177 (40.6)	283 (51.1)	131 (44.7)	39 (36.5)	0.0051
**Education, mean (SD)**	5.5 ± 3.9	5.2 ± 3.1	5.5 ± 3.8	5.8 ± 3.9	5.4 ± 4.0	4.9 ± 3.5	<.0001
**U.S. Born**	821 (58.0)	15 (60.0)	246 (56.4)	333 (60.1)	156 (53.2)	71 (66.4)	0.1215
**Current smoker**	90 (6.4)	4 (16)	32 (7.3)	38 (6.9)	10 (3.4)	6 (5.6)	0.0460
**BMI, mean (SD)**	27.5 ± 1.7	17.4 ± 1.1	22.7 ± 1.7	27.4 ± 1.4	31.9 ± 1.4	38.5 ± 3.2	0.0816
**Hypertension**	880 (62.2)	7 (28.0)	237 (54.4)	352 (63.5)	202 (68.9)	82 (76.6)	<.0001
**Heart Attack**	113 (8.0)	3 (12.0)	32 (7.3)	46 (8.3)	23 (7.9)	9 (8.4)	0.8603
**Cancer**	98 (6.9)	1 (4.0)	28 (6.4)	37 (6.7)	21 (7.2)	11 (10.3)	0.6834
**Diabetes**	452 (31.9)	4 (16.0)	108 (24.8)	177 (32.0)	107 (36.5)	56 (52.3)	<.0001
**Stroke**	84 (5.9)	2 (8.0)	21 (4.8)	32 (5.8)	23 (7.9)	6 (5.6)	0.4666
**Hip fracture**	44 (3.1)	2 (8.)	18 (4.1)	14 (2.5)	8 (2.7)	2 (1.9)	0.2793
**Depressive symptoms (CES-D ≥ 16)**	208 (14.7)	5 (20.0)	65 (14.9)	73 (13.2)	49 (16.7)	16 (15.0)	0.6335
**Any falls**	448 (31.7)	9 (36.0)	146 (33.5)	159 (28.7)	96 (32.8)	38 (35.5)	0.4017
**ADL disability**	348 (24.6)	6 (24.0)	92 (21.1)	130 (23.5)	75 (25.6)	45 (42.1)	0.0003
**MMSE, mean (SD)**	22.8 ± 5.4	20.8 ± 7.2	22.3 ± 5.5	23.1 ± 5.2	23.0 ± 5.3	22.9 ± 5.4	<.0001
**SPPB Score, mean ± SD**	6.4 ± 3.4	5.7 ± 3.6	6.8 ± 3.3	6.5 ± 3.4	6.3 ± 3.4	4.9 ± 3.6	<.0001
**Dead**	899 (63.5)	23 (92.0)	297 (68.1)	330 (59.6)	173 (59.0)	76 (71.0)	0.0002

*CES-D* Center for Epidemiologic Studies Depression Scale, *SPPB* Short Physical Performance Battery, *MMSE* Mini Mental State Examination, *BMI* Body Mass Index (Kg/m^2^), *ADL* Activities of Daily Living

Table 2 presents the results of Cox proportional hazard analysis of 12-year mortality as a function of baseline BMI categories. In the Model 1, participants in the overweight or obesity category I had decreased hazard ratio (HR) of mortality (0.82, 95% CI = 0.70–0.96 and 0.75, 95% CI = 0.62–0.91, respectively) than those in the normal weight category after controlling for all covariates. Participants in the underweight category had increased HR of mortality (1.59, 95% CI = 1.03–2.45) than those in the normal weight category after controlling for all covariates. Obesity category II/morbid obesity was not associated with mortality (HR = 1.12, 95% CI = 0.85–1.46). Female participants and those with high SPPB and MMSE scores had decreased risk of mortality, while diabetes and heart attack had an increased risk of mortality. In Model 2, without adjusting for comorbidities, overweight and obesity category I were at less risk of mortality. When we excluded those who died during the first 2 years of follow-up and those who were current smokers (Model 3), those in the overweight or obesity category I had decreased hazards of mortality.

**Table 2:** Cox proportion regression model predicting 12-year mortality as a function of baseline BMI categories among older Mexican Americans

Variables	Model 1 HR 95% CI ***N*** = 1415	Model 2 HR 95% CI ***N*** = 1415	Model 3 HR 95% CI ***N*** = 1219
**Age**	1.08 (1.06–1.10)	1.07 (1.06–1.09)	1.08 (1.06–1.10)
**Female**	0.69 (0.59–0.80)	0.67 (0.57–0.77)	0.69 (0.58–0.81)
**Married**	0.90 (0.78–1.04)	0.90 (0.78–1.04)	0.93 (0.79–1.09)
**Years of Education**	1.03 (1.01–1.05)	1.03 (1.01–1.05)	1.03 (1.01–1.05)
**U.S. Born**	1.18 (1.03–1.36)	1.18 (1.03–1.36)	1.19 (1.02–1.39)
**Smoking**	1.20 (0.92–1.56)	1.16 (0.89–1.51)	–
**BMI (Kg/m**^**2**^**) categories**			
Underweight (< 18.5)	1.59 (1.03–2.45)	1.63 (1.06–2.51)	1.50 (0.91–2.49)
Normal Weight (18.5 to < 25)	Reference	Reference	Reference
Overweight (25 to < 30)	0.82 (0.70–0.96)	0.83 (0.71–0.97)	0.75 (0.63–0.90)
Obesity category I (30 to < 35)	0.75 (0.62–0.91)	0.77 (0.64–0.94)	0.73 (0.59–0.90)
Obesity category II – Morbid Obesity (≥ 35)	1.12 (0.85–1.46)	1.21 (0.92–1.58)	1.20 (0.89–1.62)
**Hypertension**	1.00 (0.87–1.15)	–	0.93 (0.79–1.08)
**Heart Attack**	1.34 (1.05–1.69)	–	1.25 (0.95–1.64)
**Cancer**	0.99 (0.76–1.28)	–	1.04 (0.77–1.40)
**Diabetes**	1.41 (1.22–1.63)	–	1.51 (1.28–1.77)
**Stroke**	1.12 (0.84–1.49)	–	1.19 (0.87–1.63)
**Hip fracture**	1.00 (0.68–1.47)	–	1.35 (0.89–2.04)
**Depressive symptoms (CES-D ≥ 16)**	1.06 (0.87–1.28)	1.05 (0.87–1.28)	1.02 (0.82–1.27)
**Any falls**	1.01 (0.87–1.17)	1.06 (0.92–1.23)	1.03 (0.87–1.21)
**ADL disability**	1.14 (0.96–1.36)	1.11 (0.93–1.32)	1.05 (0.86–1.28)
**SPPB**	0.95 (0.93–0.97)	0.94 (0.92–0.97)	0.96 (0.93–0.98)
**MMSE**	0.97 (0.96–0.98)	0.97 (0.95–0.98)	0.96 (0.95–0.98)

Model 1 included BMI categories with all covariates. Model 2 was not adjusted for comorbidities. Model 3, participants who died the first 2 years of follow-up and those were currents smoker were excluded

*CES-D* Center for Epidemiologic Studies Depression Scale, *SPPB* Short Physical Performance Battery, *MMSE* Mini Mental State Examination, *BMI* Body Mass Index (Kg/m^2^), *ADL* Activities of Daily Living, *HR* Hazard Ratio

**Fig. 1:**
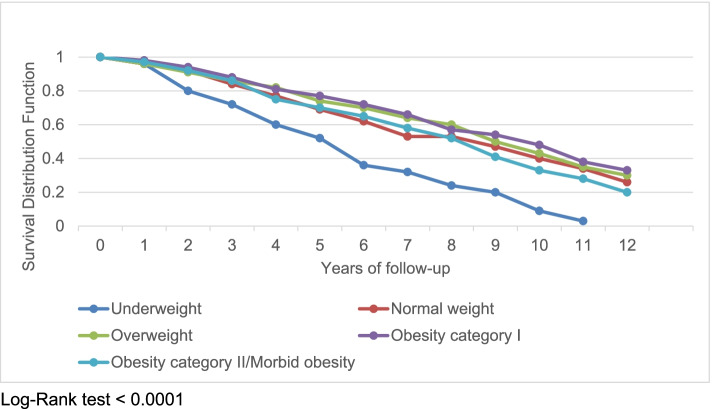
Survival curve as a function of BMI categories over 12-years of follow-up (*N* = 1415)

Figure 1 presents the survival curve of participants as a function of baseline BMI categories over 12 years of follow-up. Underweight participants had the sharpest decline in survival time over the period of follow-up.

## Discussion

This study examined the effect of baseline BMI categories on 12-year mortality among Mexican American men and women aged 75 years and older. We found that compared to those in the normal weight category, overweight or obesity category I had an 18 and 25% decreased risk of mortality, respectively. Those in the underweight or obesity category II/morbid obesity had a 59 and 12% increased risk of mortality than those in the normal weight category. We found decreased risk of mortality among females and those who had higher scores on the MMSE and SPPB tests. This study showed the protective effect of overweight and obesity on mortality in Mexican Americans above 75 years of age, which might have implications when treating older adults with overweight and obesity.

Little has been written about BMI and mortality in the oldest old, especially among Mexican Americans. Our findings are similar to prior studies. For example, our findings are similar to those reported in the study of Finnish older adults above 70 years of age where overweight and obesity was associated with 23 and 24% decreased risk of mortality, respectively, over 33 years of follow-up [16]. Our results were also consistent with the limited number of studies that included participants over 80 years of age which showed overweight [15, 17, 18] and obesity [15, 18] to be associated with lower mortality and underweight [15, 18] with higher mortality. These studies included Polish, Swedish, or Chinese men and women. Dahl et al. found in a cohort of Swedish older adults aged 70 to 95 years that those in the overweight group had a 20% decreased risk for mortality compared to the normal group over 4 years of follow-up for those younger than 80 years and 2 years of follow-up for those over 80 years [17]. Wang et al. using the Chinese Longitudinal Healthy Longevity Survey (CLHLS) found that overweight and obese participants aged 80 years and older had a 11 and 9% decreased risk of mortality, respectively, compared with the normal weight group over 3 years of follow-up [18] Takata et al. found in a cohort of Japanese older adults aged 80 and older that the most-lean group (BMI < 19.5) had the highest all-cause mortality while the group with BMI 22.5 to < 23.8 had the lowest all-cause mortality [28]. Although our results are consistent with these studies, they include different ethnicities of participants which makes comparison difficult due to ethnic differences in body composition. Rolland et al. found in a cohort of French older women aged 75 years and older that mortality risk was higher in participants with BMI ≤ 24.6 at 12-years of follow-up [29]. Although we found similar results, this study used BMI as a continuous variable, had a different ethnic demographic, and included women only.

There are several possible mechanisms through which higher adiposity may confer lower mortality risk in the very old and oldest-old. First, adiposity in older adults may be protective from malnutrition by allowing an escape from a negative energy balance due to a greater metabolic reserve [30–32]. Second, obesity has been associated with increased bone density in older adults [31], which can offer protection from osteoporotic fractures through a fat cushioning effect for surrounding areas, like the hip, which are prone to fracture after a fall [4]. Third, overweight and obesity can modulate progression of heart failure [31, 32]. Studies have found overweight and obesity offer a better prognosis in chronic heart failure, possibly due to lower atrial naturetic peptide levels, lower sympathetic activation, and decreased reponse to activated renin-angiotensin-aldosterone system (RAAS) [33]. Fourth, adiposity in the older adults may provide better antioxidant defense [4]. Sarcopenia from weight loss results in loss of muscle mass in older adults [4]. According to Oreopoulos et al. sarcopenia reduces oxidative metabolism in the skeletal muscle which could increase oxidative stress and inflammation [4]. Lastly, reverse causality may also be playing a role in adiposity conferring a protective effect on mortality. Garcia et al. examined BMI and mortality in a U.S. cohort over 5 years of follow-up by excluding smokers and those with pre-existing conditions [34]. Exclusion of these individuals resulted in lower mortality risk in the underweight than it would have been included [34].

This study has some limitations. First, co-morbidities were assessed through self-reports. However, studies have shown agreement between self-report and comorbid conditions or diseases as well as medical events [35]. Second, participants excluded from the study were less healthy compared to those included, which might have resulted in underestimating the relationship between BMI and mortality. However, when we excluded those who died in the first 2 years of follow-up and those who were current smokers, the relationship between BMI and mortality did not change. Third, we did not analyze BMI categories change over time. However, previous studies have shown that increasing BMI was not associated with higher risk of mortality while decreasing BMI associated with higher risk of mortality [36–38]. Fourth, maybe the BMI value is overestimated due to height loss in older adults due to decrease vertebral bone mineral density [31]. We used BMI instead of other anthropometric measurements because it has been found that adjusting for height loss only has a slight effect on BMI when examining the association with mortality and healthcare cost estimates [39–41]. Fifth, our sample is not generalizable to the entire Hispanic population in the United States because it consisted only of Mexican Americans living in the Southwestern US. However, findings from the National Health Interview Survey that included only Hispanics showed that underweight was associated with increased mortality while those in the overweight or obesity category I were at increased mortality over 8-years of follow-up [42]. Similarly, findings from the National Health and Nutrition Examination Survey that analyzed only Mexican Americans showed BMI was not significantly associated with mortality [43]. Despite these limitations, our study has several strengths including the use of a large population-based sample of community dwelling of Mexican Americans aged 75 years and older, follow-up data over 12 years which is longer than the studies conducted in other race/ethnic groups, and the inclusion of both females and males.

## Conclusions

The results of our study suggest the protective effect of overweight and obesity category I against mortality in Mexican Americans aged 75 years and older. At 12 years of follow-up, Mexican Americans aged 75 years and older with overweight or obesity category I had statistically decreased risk of mortality compared to normal weight. While participants with underweight had a statistically increased risk of mortality compared to normal weight. Our study has many implications for the care of older Mexican American adults in the group of very old and oldest-old. BMI should be carefully monitored by healthcare providers when caring for this population. Based on the height of the patient, an appropriate weight range can be calculated, suggested, and monitored. Appropriate prevention and intervention programs based on BMI maybe developed to maintain a healthy weight. With our study finding underweight having an increased risk of mortality, our public health recommendation for the very old and oldest old is to avoid drastic weight loss in order to not be in the underweight category. Instead, their weight should be maintained, particularly in the overweight or obesity type 1 categories. Providers should be cautious in recommending weight loss in the very old and oldest old [31]. More studies examining BMI and mortality must be done in this population to further support our conclusions.

## Data Availability

The datasets generated during and/or analyzed during the current study are available at https://www.icpsr.umich.edu/icpsrweb/NACDA/series/546.

## Abbreviations

BMI: Body Mass Index
MMSE: Mini-Mental-State-Examination
SPPB: Short Physical Performance Battery
ADL: Activities of Daily Living
HR: Hazard Ratio
HEPESE: Hispanic Established Population for the Epidemiologic Study of the Elderly
CDC: Centers for Disease Control and Prevention
